# Transcriptome analysis of alcohol-treated microglia reveals downregulation of beta amyloid phagocytosis

**DOI:** 10.1186/s12974-018-1184-7

**Published:** 2018-05-14

**Authors:** Sergey Kalinin, Marta González-Prieto, Hannah Scheiblich, Lucia Lisi, Handojo Kusumo, Michael T. Heneka, Jose L. M. Madrigal, Subhash C. Pandey, Douglas L. Feinstein

**Affiliations:** 1 Department of Anesthesiology, University of Illinois at Chicago, Chicago, IL 60612 USA; 2 Department of Pharmacology, University Complutense, Centro de Investigacion Biomedica en Red de Salud Mental (CIBERSAM), Madrid, 28040 Spain; 3 Department of Neurodegenerative Disease and Geriatric Psychiatry, University of Bonn, 53127 Bonn, Germany; 4 Institute of Pharmacology, Catholic University Medical School, 00168 Rome, Italy; 5 Center for Alcohol Research in Epigenetics, Department of Psychiatry, University of Illinois at Chicago, Chicago, IL 60612 USA; 6 Department of Veterans Affairs, Jesse Brown VA Medical Center, Chicago, IL 60612 USA

**Keywords:** Alcohol, Phagocytosis, Complement, Alzheimer’s disease, Amyloid, Microglia

## Abstract

**Background:**

Microglial activation contributes to the neuropathology associated with chronic alcohol exposure and withdrawal, including the expression of inflammatory and anti-inflammatory genes. In the current study, we examined the transcriptome of primary rat microglial cells following incubation with alcohol alone, or alcohol together with a robust inflammatory stimulus.

**Methods:**

Primary microglia were prepared from mixed rat glial cultures. Cells were incubated with 75 mM ethanol alone or with proinflammatory cytokines (“TII”: IL1β, IFNγ, and TNFα). Isolated mRNA was used for RNAseq analysis and qPCR. Effects of alcohol on phagocytosis were determined by uptake of oligomeric amyloid beta.

**Results:**

Alcohol induced nitrite production in control cells and increased nitrite production in cells co-treated with TII. RNAseq analysis of microglia exposed for 24 h to alcohol identified 312 differentially expressed mRNAs (“Alc-DEs”), with changes confirmed by qPCR analysis. Gene ontology analysis identified phagosome as one of the highest-ranking KEGG pathways including transcripts regulating phagocytosis. Alcohol also increased several complement-related mRNAs that have roles in phagocytosis, including C1qa, b, and c; C3; and C3aR1. RNAseq analysis identified over 3000 differentially expressed mRNAs in microglia following overnight incubation with TII; and comparison to the group of Alc-DEs revealed 87 mRNAs modulated by alcohol but not by TII, including C1qa, b, and c. Consistent with observed changes in phagocytosis-related mRNAs, the uptake of amyloid beta_1–42_, by primary microglia, was reduced by alcohol.

**Conclusions:**

Our results define alterations that occur to microglial gene expression following alcohol exposure and suggest that alcohol effects on phagocytosis could contribute to the development of Alzheimer’s disease.

**Electronic supplementary material:**

The online version of this article (10.1186/s12974-018-1184-7) contains supplementary material, which is available to authorized users.

## Background

Microglial activation occurs in a variety of neurological conditions and diseases, and increasing number of studies show that microglial activation contributes to the neuroinflammation associated with chronic alcohol exposure and withdrawal. Studies indicate that innate immune signaling plays a role in alcohol addiction, and genes implicated in neuroinflammatory processes accompanying alcoholism are expressed in microglia [1–3]. Supporting a role for microglial activation are studies showing that treatment with minocycline, which selectively reduces microglia activation, reduces alcohol intake as well as acute actions of ethanol on sedation and motor impairment [4, 5], and similarly, treatment of mice with tigecycline, another tetracycline derivative, reduced alcohol consumption and clinical features of alcohol consumption in mice [6]. There are also increasing studies demonstrating that microglia can be directly activated by alcohol leading to increases in inflammatory factors including cytokines, chemokines, transcription factors, and their receptors [1, 2, 7–13]. In many cases, the effects of alcohol were shown to involve activation of the Tlr4 receptor [9, 10, 12, 14–17] which can directly activate inflammatory transcription factor NFkB and subsequently increase of inflammatory gene expression. Alcohol has also increased the expression of TLRs, in both the liver [18] and the CNS [19, 20]. Other studies have shown that alcohol also increases expression and acetylation of HMGB1 which can bind to TLRs and thereby induce inflammatory gene expression [3, 19].

Despite the above studies that focused on specific genes or categories of genes, there are few papers which provide characterization of the microglial transcriptome following alcohol treatment or consumption. Transcript profiling has been carried out using RNA from whole brain [4], amygdala [21], prefrontal cortex [22], and nucleus accumbens [23] from alcohol-fed mice. While pathway analysis identified many microglial associated functions, only a single study has directly examined the transcriptome of microglia acutely isolated from cortex of alcohol-fed mice [24]. In that study, over 400 transcripts were identified in the microglial that were not present in pre-frontal cortical RNA [22], suggesting that cell type enrichment is necessary to fully characterize the effects of alcohol on the microglial transcriptome.

While microglial activation and increased pro-inflammatory cytokine and chemokine expression can initiate or exacerbate ongoing pathology, microglia also perform beneficial actions that limit damage in neurological diseases and conditions. Microglial-mediated processes are a key determinant to the accumulation of amyloid deposits in AD (and its mouse models), playing roles in amyloid degradation (by metalloproteases including neprilysin and insulin degrading enzyme), in removal of amyloid by phagocytosis, and by initiation and growth of plaques (involving seeding by microglial inflammasome activation) [25]. Dysregulation of these processes will alter the balance between amyloid production and clearance, with the net result of increased amyloid burden. In Alzheimer’s disease (AD), microglial phagocytosis of oligomeric and aggregated forms of amyloid beta (Aβ) is one of the key means by which amyloid burden is limited [26, 27]. Several studies suggest that alcohol may be a risk factor for AD [28–31], and there are also reports that alcohol increases amyloid processing and deposition [32–34]. However, whether alcohol influences the ability or efficacy of microglial cells to internalize Aβ has not been examined, although several studies have shown that peripheral macrophages have reduced phagocytotic activity after alcohol treatment [35, 36].

In the current study, we evaluated the acute effects of alcohol on inflammatory responses in primary rat microglial cells using RNAseq analysis to define the changes in gene expression due to alcohol. Our results define alterations in microglial mRNA expression following exposure to alcohol and suggest that alcohol consumption may represent a risk factor for development of amyloid burden.

## Methods

### Primary microglial cells

All studies with animals were approved by the UIC Institutional Animal Care and Use Committee. Primary mixed glial cells were prepared from the frontal cortices of grouped male and female post-natal day 2 Sprague Dawley rats from the same litter [37]. In brief, cerebral cortices were cleaned from all meninges, digested in trypsin, and dissociated into single cell suspension by trituration through syringes. The cells were plated onto poly-l-lysine-coated flasks and grown in Dulbecco’s modified Eagle’s media (DMEM) supplemented with 10% heat inactivated fetal bovine serum (FBS) and 1% antibiotics (P/S; penicillin/streptomycin, Gibco, ThermoFisher, Waltham, MA, USA). The next day, cells were washed with PBS to remove debris, and the media were changed twice per week. After 7–10 days, loosely attached microglia were removed from underlying astrocytes by shaking flasks at 220 RPM for 30 min at 37 °C. Cells were collected, replated into dishes in DMEM containing 10% FBS and 1% P/S, and allowed to adhere overnight. The next day, the media were changed to serum free DMEM with 1% N-2 supplement.

### Alcohol and cytokine treatment

Microglia were exposed to ethanol in incubator chambers (Modular Incubator Chambers MIC-101, Billups-Rothenberg Inc. Del Mar, CA) containing either 100 mL ddH2O alone or 75 mM ethanol. Ethanol was added directly to the cell media to bring the final concentration to 75 mM. The chambers were flushed with 5% CO_2_, 21% O_2_, balanced nitrogen mixture from a compressed air tank at 0.5 psi for 4 min, and then incubated at 37 °C for 24 h. Where indicated, a mixture of pro-inflammatory cytokines (“TII”, TNFα, 10 ng/ml; IL-1β, 10 ng/ml; and IFNγ, 10 IU/ml) dissolved in cell culture media was added to the cells to induce an inflammatory response; control cells received the equivalent volume of cell culture media.

### Phagocytosis assay

Phagocytosis was assessed in rat primary microglial cells. For this, the cell culture media was first changed to serum-free DMEM supplemented with 1% N-2 supplement (Gibco). After 24 h, cells (3.5 × 10^5^ cells/well) were incubated under control conditions or in medium containing 75 mM EtOH, TII, or TII with 75 mM EtOH. After 24 h, the medium was replaced with fresh medium (control or containing 75 mM EtOH) supplemented with 6-carboxyfluorescein (FAM)-labeled Aβ_1–42_ (0.5 μM; Anaspec, Fremont, CA, USA). The Aβ peptide was dissolved in DMSO to obtain a 0.1-mM stock, diluted into DMEM to a final concentration of 500 nM, then incubated at 37 °C for 1 h to promote aggregation. Cells were incubated for indicated times, followed by one washing with PBS to remove Aβ, then harvested using 0.5% Trypsin (Gibco). Blocking solution containing PBS and FBS (1:1 ratio) was applied for 10 min on ice. Cells were collected, resuspended in 400 μl ice cold FACS solution (PBS supplemented with 2% FCS), and measured by flow cytometry using the Gallios software (Beckman Coulter’s). Phagocytosis was analyzed and quantified for total uptake and for the percentage of cells with internalized Aβ, using Flowing Software (University of Turku, Finland).

### Nitrite production

Inflammatory activation of microglia was assessed indirectly as the production of nitrites in the cell culture media, an index of the induction of nitric oxide synthase type 2, measured using Griess reagent. Background values were obtained using  media only and were subtracted from values obtained using cells.

### RNA isolation

RNA was isolated using Direct-zol RNA MicroPrep (Zymo Research, Irvine, CA, USA) according to instructions. The resulting RNA quality was determined using the 4200 TapeStation Instrument (Agilent, Santa Clara, CA), and all samples had RNA integrity numbers (RIN) above 8.

### Library generation

Illumina compatible libraries were prepared from RNA using QuantSeq 3′ mRNA-seq Library Prep Kit FWD for Illumina (Lexogen GmbH, Wien, Austria) according to the manufacturer’s instructions. In brief, library generation was initiated by oligo-dT priming and first-strand synthesis. After RNA removal, libraries were subjected to random-primed second-strand synthesis. Illumina specific linker sequences are added by the primer, and the resulting double-stranded cDNA purified with magnetic beads. An additional 12 cycles of PCR amplification were carried out in order to introduce barcodes and to generate sufficient amounts of DNA required for cluster generation. After final purification, libraries were measured on TapeStation and Qubit (ThermoFisher, Waltham, MA) to determine quantity and size. The resulting libraries were on average 400-bp size with an average insert size of 270 bp. The method does not require prior poly(A) enrichment or ribosomal RNA depletion. ERCC (External RNA Controls Consortium) RNA Spike-In Mix (Cat# 4456740 Thermo Fisher Scientific, Waltham, MA) was added to the RNA before library preparation to allow inter-sample normalization and control for variabilities.

### RNA sequencing and analysis

Barcoded libraries were pooled and sequenced on Illumina NextSeq system (Illumina, San Diego, CA, USA) producing about 500 M reads of non-paired 75-nt sequence. Up to 32 barcoded samples were pooled together producing on average 12 M reads per sample. RNAseq analysis was carried out using the BaseSpace platform from Illumina. The RNAseq-generated FASTQ files were aligned to the USCSrn5 *Rattus norvegicus* reference genome with STAR aligner [38] with allowed mismatches set to 14. Differentially expressed (DE) mRNAs were determined using the DeSeq2 package based on the negative binomial distribution and a false discovery rate of 0.1% [39]. In brief, paired RNAseq data for each transcript are compared using Wald testing which is a more powerful method than others to detect significant differences in low expression transcripts [40]; those with Wald *p* values < 0.05 are ordered, and an adjusted *p* value is then determined using Benjamini-Hochberg approach to minimize false discovery to 0.1% or less. This method does not take into consideration the magnitude of the difference in expression. Functional and pathway analysis were performed using DAVID [41] and GO Consortium [42, 43] platforms.

### Quantitative real-time PCR

Whole cell RNA (1 μg) was converted to cDNA using the High Capacity cDNA Reverse Transcription Kit (Applied Biosystems cat #4368814, ThermoFisher, Waltham, MA, USA). The cDNA was amplified using FastStart Universal SYBR Green Master mix (Applied Biosystems, cat #04913914001, Foster City, CA, USA) in a Corbett RotoGene real-time PCR machine (Qiagen, Germantown, MD). The relative levels of mRNA were calculated from threshold take-off cycle number and normalized to values measured for β-actin in the same samples.

### Data analysis

Data are presented as mean ± SEM of at least three independent experiments. qPCR data were compared using Student’s *t* tests. Nitrite data were compared using one-way parametric ANOVA and Tukey’s post hoc comparisons. Phagocytosis data were analyzed for Gaussian distribution; all data passed normality test so comparisons were performed with one-way ANOVA and Tukey’s post hoc comparisons.

## Results

### Alcohol increases microglial inflammatory activation

**Fig. 1:**
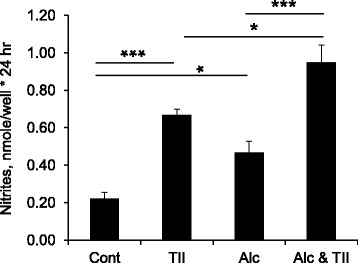
Alcohol increases microglial inflammatory activation. Primary rat microglia were incubated overnight with nothing (control), with TII to induce an inflammatory response, with 75 mM ethanol (Alc), or with TII and ethanol (Alc and TII). The next day, nitrite production was measured using Griess reagent. The data is the mean ± sem of *n* = 6–15 replicates done in 3 independent studies. *P < 0.05; ***P < 0.0001; one-way ANOVA, Tukey’s post hoc analysis

Enriched (> 95%) primary microglia were prepared by the shake-off method from postnatal day 2 rat cortical mixed glial cultures, then incubated with 75 mM ethanol, with or without TII to induce inflammatory activation as assessed by measurement of nitrite levels in the culture media. Under these conditions, ethanol alone increased nitrite levels about twofold above control values (Fig. 1). Incubation with TII increased nitrite production about threefold over control, and that was further increased (to about fourfold control values) when ethanol was present.

### Alcohol-dependent changes in microglial mRNA

**Fig. 2:**
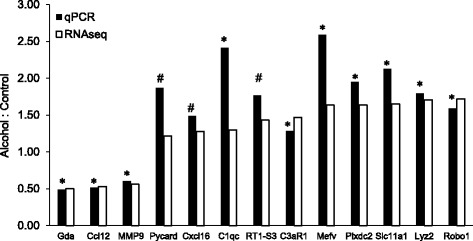
Validation of RNAseq results. Reverse transcriptase quantitative PCR (qPCR) was used to measure mRNA levels of the indicated Alc-DEs. qPCR was carried out in samples from microglia incubated for 24 h with nothing (control, *n* = 3) or 75 mM ethanol (*n* = 3), and results normalized to values measured for β-actin in the same samples. The *y*-axis shows the ratio of the average mRNA level measured in the ethanol versus the control samples (filled bars) and is plotted next to the fold-difference calculated from RNAseq data (open bars). For qPCR results, *P < 0.05; ^#^*P* < 0.10 control vs alcohol. For the RNAseq data, all DEs were found significantly different using Deseq2

RNAseq analysis was used to identify mRNAs regulated by exposure to alcohol. Of 17,327 genes in the rat reference genome, 15,062 mRNAs were identified having over 0.1 FPKM (Additional file 1: Table S6); of those, 312 were differentially expressed (DE) following ethanol treatment (“Alc-DEs”; Additional file 1: Table S1). The majority of Alc-DEs (230) were increased, while 82 were decreased by ethanol. Of those, 274 were changed by at least 20% and 19 changed by at least 50%. Gda (guanine deaminase) was the mRNA most decreased (to about 50% of control values), and Robo1 (roundabout guidance receptor 1) and Plxdc2 (plexin domain containing 2) were the most increased (to 170% of control values) by alcohol. Quantitative reverse-transcription PCR (qPCR) of the mRNAs showing the largest changes, as well as of several other mRNAs selected for having important functional consequences, validated RNAseq results (Fig. 2).

To compare microglial responses to alcohol to those elicited by an inflammatory stimulus, we carried out RNAseq on RNA from TII-treated microglia. In contrast to alcohol, there was a dramatic alteration in mRNA expression due to this robust stimulus. Using the same criteria (*P* values adjusted for an FDR of < 0.1%), we identified 3082 mRNAs modified by TII (“TII-DEs”; Additional file 1: Table S2). Of those, 1266 were changed by over 50%; 43 mRNAs increased over 10-fold; and 21 decreased by at least 10-fold. To determine how alcohol influences microglial gene expression in the context of inflammation, we carried out RNAseq of mRNA from cells treated with TII together with ethanol. A total of 3552 mRNAs were identified (“AlcTII-DEs”, Additional file 1: Table S3), slightly more than that due to TII alone. Of those, 1432 were changed by over 50%, 53 mRNAs were increased over 10-fold, and 25 were decreased by at least 10-fold.

**Fig. 3:**
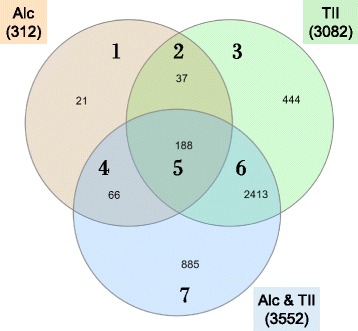
Overlap of identified DEs. A Venn diagram illustrating overlap of Alc-DEs (*n* = 312; orange, regions 1, 2, 4, and 5), TII-DEs (*n* = 3082; green, regions 2, 3, 5, and 6) and AlcTII-DEs (*n* = 3552; blue, regions 4, 5, 6, and 7). The full list of the DEs in each group is provided in Additional file 1: Tables S1-S4

Comparison (Fig. 3 and Additional file 1: Table S4) of Alc-DEs (regions 1, 2, 4, and 5) to TII-DEs (regions 2, 3, 5, and 6) shows that 225 of the 312 Alc-DEs are present in both groups (regions 2 and 5). The remaining 87 Alc-DEs (regions 1 and 4) are not induced by a robust inflammatory stimulus; therefore, these represent a non-canonical type of microglial activation; we refer to this group as Alc*-DEs. Further comparison to the AlcTII-DE group shows that 21 DEs (“AlcOnly-DEs”, region 1) are found only in the Alc-DE group. Comparison of AlcTII-DEs to TII-DEs shows considerable overlap (2601 DEs, regions 5 and 6) between these two groups; as well as an additional 951 (“AlcInf-DEs”, regions 4 and 7) that are not detected in the TII group. Of those 951 AlcInf-DEs, 885 (“Alc*TII-DEs”, region 7) are increased by alcohol only in the context of inflammation but not by alcohol or by TII alone.

Gene Ontology analysis (DAVID) identified five KEGG pathways enriched in the 312 Alc-DE group (Additional file 1: Table S5A), with one of the highest categories being phagosome containing 15 transcripts (Atp6ap1, C3, Calr, Coro1a, Ctss, Cyba, Fcgr1a, Itgb5, Ncf1, RT1-A1, RT1-CE14, RT1-N3, RT1-S3, Tuba1b, and Tlr2). In addition, a Gene Ontology Consortium analysis [42, 43] identified a ninefold enrichment in transcripts involved in regulation of phagocytosis including Psap, Pycard, CD300lf, Slc11a1, Mfge8, and Sirpa. DAVID analysis of the 21 AlcOnly-DEs did not identify any enriched GO terms or KEGG pathways likely due to the small group size. However, four members (C1qb, Ifih1, Ly86, and Tmem30a) play roles in the innate immune system and Tlr4 signaling, and three members (Laptm5, Map1Lc3a, and Uba7) are involved in protein degradation. In the group of 87 alcohol-specific Alc*-DEs, only endoplasmic reticulum (ER) was identified as an enriched cytosolic component. However, this group includes all 3 isoforms of complement protein C1q, as well as 7 other mRNAs (Oas1b, Rtp4, Ifih1, Pou2f2, Kdelr1, Coro1a, and Ddx58) involved in immune effector responses. There are four KEGG pathways identified in the group of 885 Alc*TII-DEs (Additional file 1: Table S5B), three of which involve RNA handling (ribosome, spliceosome, and transport) and one involves Salmonella infection. Interestingly, while levels of 14 of the 18 mRNAs encoding nuclear ribosomal subunits were decreased, the levels of 7 of the 8 mRNAs encoding mitochondrial ribosomal subunits were increased.

### Alcohol reduces microglial phagocytosis

**Fig. 4:**
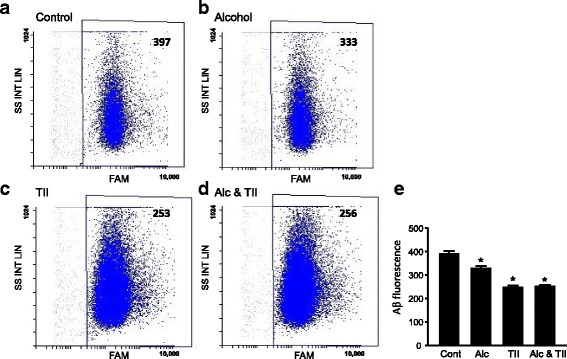
Alcohol reduces amyloid beta phagocytosis in rat microglia. Primary rat microglia were incubated overnight with **a** nothing (control), **b** 75 mM ethanol, **c** TII, or **d** 75 mM ethanol and TII. The next day, the cells were assayed by flow cytometry for phagocytosis of FAM-labeled oligomeric Aβ_1–42_. **e** Average internalized FAM fluorescence per cell. Data is from 3 different batches of microglial cells totaling 9197 (controls), 8976 (alcohol), 24,588 (TII), and 28,773 (Alc * TII) events. *P < 0.005 versus control cells, one-way ANOVA, Tukey’s post hoc analysis

Since phagocytosis was one of the highest KEGG pathways identified in the Alc-DE group, we tested if alcohol influenced microglial phagocytosis. In primary rat microglial cells, overnight exposure to alcohol reduced phagocytosis of fluorescently labeled oligomeric Aβ_1–42_ (Fig. 4a, b). In these cells, incubation with TII cytokines also reduced Aβ phagocytosis compared to control cells, but that reduction was not affected by the presence of alcohol (Fig. 4c, d). Quantitation of the average amount of Aβ internalized over 45 min shows a significant 16% reduction due to alcohol and a 37% reduction due to TII (Fig. 4e).

## Discussion

Several studies have examined the effects of alcohol consumption on neuroinflammatory gene expression. Transcript profiling has been carried out using RNA from the whole brain of mice fed alcohol 4 h per day for 4 days [4]; from amygdala (AMY) after 30 days drinking using a 2-bottle choice [21]; from prefrontal cortex (PFC) after chronic (30 day), chronic intermittent (every other day for 29 days) or drinking in the dark (DID, 4 h in the dark for 36 days) [22]; and from PFC, Nucleus Accumbens (NAC), and AMY after 4 weekly cycles of chronic intermittent drinking [23]. In these studies, pathway analysis identified enrichment for many microglial mRNAs and networks, consistent with a role for microglia activation by alcohol. In microglia acutely isolated from prefrontal cortex of mice after alcohol consumption for 60 days in an every-other day drinking paradigm [24], 1010 Alc-DEs were identified in the microglial samples, compared to 2461 in total homogenates. Of the 1010 DEs, 846 were unique to microglia and not detected in the total homogenates. Similar to our findings, the largest differences were less than twofold for the DEs. Overall, comparison of the 846 alcohol-induced microglial specific changes to the 312 Alc-DEs identified in the current study showed only 28 overlapping mRNAs. Whether this large difference reflects the long duration of the in vivo paradigm compared to a relatively brief 24 h period of in vitro exposure or to differences between acutely isolated microglia to primary microglial prepared from mixed glial cultures is not known.

In our study, the average change in the 313 Alc-DEs was 16%, ranging from a 50% decrease to a 72% increase. While modest, these changes are consistent with those reported for microglial transcripts in other studies. In enriched microglia isolated from CTX of alcohol-fed rats [24], the majority of DEs had expression changes between log2 of 0.125 (9%) and 0.375 (30%); changes for the total cortical homogenates were somewhat greater, ranging from log2 of 0.10 (7%) to about 0.5 (50%). In a study of microRNAs, the analysis for DEs used cutoff thresholds of 5% [23], and in a study of synaptosomal transcripts [21], the majority of transcripts showed fold changes on the order of 20% or less. These changes are consistent with reports that modest differences in alcohol-induced gene expression are common for CNS [44]. These observations suggest that modest changes in a set of functionally related mRNAs (and associated proteins) can exert as significant an effect as does a robust change in expression of a single transcript.

In addition to identification of genes altered by alcohol use, studies have been carried out in rodent [45–48] and human samples to identify gene expression patterns that correlate with alcohol use or preference [49–52]. A comparative network analysis of RNAseq data from rats bred for either high (SHR) or low (BN-LX) alcohol preference, and by including correlation to phenotypic data from a recombinant inbred population, allowed identification of candidate genes associated with alcohol consumption [47], many of which were known to be expressed in microglia (or astrocytes). Analysis of RNA from brain regions of alcoholic compared to non-alcoholic patients have identified patterns of gene expression that discriminate between groups [51, 52]. In the nucleus accumbens of patients with alcohol dependency, over 4500 transcripts were identified as being differentially expressed, which could be clustered into 24 mRNA co-expression networks of which 6 were significantly correlated with dependency [52] and 4 were enriched for glial transcripts. However, in these cases, it is not known if expression changes were direct consequences of alcohol, or secondary to alcohol-induced damage.

In our studies, pathway analysis using DAVID [41] and GO Consortium [42, 43] platforms identified 5 KEGG pathways enriched in the 312 Alc-DE group, with phagosome being one of highest including transcripts for Atp6ap1, C3, Calr, Cd300lf, Coro1a, Ctss, Cyba, Fcgr1a, Itgb5, Mfge8, Ncf1, Psap, Pycard, RT1-A1, RT1-CE14, RT1-N3, RT1-S3, Sirpa, Slc11a1, and Tlr2. Many of these genes regulate the processes of phagocytosis and degradation (Table 1). In addition, we found that alcohol increased levels of several complement transcripts, including C1qa,b, and c; C3; and C3aR1, and moreover that the C1q variants were not increased by TII alone. This is consistent with studies showing that alcohol increases complement proteins, including C1q, C3a, C5a, C3aR, and C5aR, in liver [65, 66] and adipose tissue [67]. Since microglial complement activation can cause neuronal damage [68], these findings suggest that C1q induction in the brain could contribute to alcohol-induced neuropathology. While this may be mediated through activation of the complement pathways, observations that several complement proteins, including C1q and C3b promote phagocytosis [69, 70] and that CR3 regulates amyloid clearance [71–74], suggests that alcohol-induced changes in complement expression may also regulate microglial phagocytosis of amyloid.

**Table 1:** Phagosome-related mRNA enriched in alcohol-treated microglia

Symbol	Name and function
Coro1a	Coronin1a
	Cell membrane associated protein that interacts with actin filaments to facilitate cell motility, endocytosis, and phagocytosis. Loss of Coro1a expression or ability to bind to F-actin impairs these processes [53].
ATP6ap1	ATPase H+ transporting accessory protein 1
	Component of the H+ transporting vacuolar ATPase present in phagosomes [54]
Fcgr1a	Fc fragment of IgG receptor Ia
	Complexes with leukotriene B4 receptor in lipid rafts, enhances macrophage anti-microblial actions [55]
RT1	Proteins of the MHC class I family, involved in antigen presentation.
Ctss	Cathepsin S
	Peptidase present in phagolysosomes where it degrades various target proteins [56]
Cyba	Cytochrome b-245 alpha chain
Ncf1	Neutrophil cytosolic factor 1
	Components of the NADPH Oxidase complex, present in phagolysomes.
Slc11a1	Solute carrier family 11 member a1, also referred to as Nramp1
	Transmembrane phagosomal divalent cation transporter [57]
Mfge8	Ligand milk fat globule EGF factor-8
	Ligand for Itgb5 (integrain subunit beta 5) required for activation of several pathways, including MerTK activation and F-actin recruitment, involved in clearance [58]
Psap	Prosaponin
	Precursor of saposins A, B, C, and D, which have roles in lysomal degradation pathways [59]
Sirpa	Signal regulatory protein alpha
	Macrophage receptor for CD47 which is a broad inhibitor of phagocytosis [60, 61]
Calr	Calreticulin
	When present on the cell surface acts as a signal to activate macrophage phagocytosis [62]
CD300lf	Member of the CD300 receptor family
	Roles in activating macrophage engulfment by phosphatidylserine signaling [63]
Pycard	PYD And CARD Domain Containing, also referred to as ASC (Apoptosis-Associated Speck-Like)
	Component of the NLRP3 inflammasome, recently shown to play a role in amyloid deposition. [64]

Our results demonstrate that microglial phagocytosis of Aβ_1–42_ is significantly suppressed following 1-day exposure to 75 mM ethanol. This dose of ethanol is in the high range and is attained in human following binge drinking or in heavy drinkers. Similar doses have previously been used to study phagocytosis in vitro [33, 36]. Suppressive effects of alcohol on phagocytosis have previously been reported in studies examining macrophages (see [35] for review). Alcohol reduces uptake of *Pseudomonas aeruginosa* [36], and of *Candida albicans* [75], and inhibition can be seen as soon as 1 h after treatment with ethanol [76]. In contrast to macrophages, there are limited studies of the effects of alcohol exposure on microglial phagocytosis. In neonatal mice [77], acute binge-like alcohol exposure induced microglial activation and phagocytosis of damaged neurons, suggesting that acute ethanol exposure could be protective during early development. It was also shown using a similar acute exposure model, that although activated microglia were observed near to dead cells in the cortex, that apoptotic bodies accumulated, interpreted that the rate of cell death exceeded microglial clearance capacity [78]. In embryonic stem cell-derived microglia [79], 48-h exposure to 100 mM ethanol decreased phagocytosis of fluorescently labeled *E. coli* particles by 15% compared to control cells. These findings are consistent with the ability of alcohol to inhibit microglia in vivo.

In our studies, alcohol exposure reduced phagocytosis of Aβ with no effect on uptake of polystyrene beads (unpublished findings, DLF). Aβ phagocytosis is regulated by various proteins several of which were identified as being induced by alcohol treatment. In primary microglia, activation of SIRPb1 (signal regulatory protein beta-1) increased phagocytosis of fibrillary Aβ, as well as of microsphere beads [80]. In contrast, inhibition of CLIC1 (chloride intracellular ion channel) increased Aβ phagocytosis, possibly via suppression of pro-inflammatory cytokine or iNOS induction [81], while inhibition had no effect on bead uptake. The basis for this difference is not known, but may be related to the ability of Aβ but not polystyrene beads to induce microglia cytokine production, which in turn regulates phagocytotic activities.

Alcohol consumption is generally considered a risk factor for dementia, although there are some inconsistencies which may depend upon age, gender, amounts consumed, and numerous other genetic and environmental factors. Indications that alcohol can worsen or accelerate dementia may contribute to the risk or progression AD; however, those studies do not address if alcohol specifically modifies AD pathogenesis. Analysis of 125 brain autopsy samples showed that, as expected, higher Aβ immunoreactivity (Aβ-IR) was associated with increased age and ApoE4 allele [82]. However, Aβ-IR was significantly inversely associated with beer drinking with an odds ratio close to 0.35, although the significance was reduced when stratified for age and ApoE4 [82]. Epidemiological studies report a reduction in AD prevalence due to low alcohol ingestion, and protective effects in those having moderate consumption [30]. Similarly, a prospective study of over 3000 subjects over 3 years found that light to moderate alcohol consumption reduced the risk of overall dementia by about 30% [83]. In contrast, a recent review of the literature to determine if alcohol consumption is a risk factor for AD concluded that alcohol use causes cognitive impairment by contributing to the neurodegenerative processes [84]. A systematic review concluded that there is as yet no consensus on this issue and that despite several studies, alcohol should not be considered methods to reduce AD risk [85].

In contrast, evidence that alcohol can increase amyloid levels comes from several studies. In vitro, low ethanol exposure, equivalent to moderate alcohol usage, decreased Aβ binding to neurons and thereby reduced neurotoxic actions of Aβ [86] which may account for protective actions at low to moderate doses. Exacerbation of AD pathogenesis by alcohol has been reported using both in vitro and in vivo studies. In human, SK-N-MC neuroblastoma cells ethanol upregulated BACE1 expression and Aβ production, as well as increased reactive oxygen species (ROS) production, cyclooxygenase-2 (COX-2) expression and PGE_2_ production [32]. Ethanol exposure of mice for 4 weeks increased APP levels and BACE1 expression, promoted Aβ production, increased plaque deposition, and worsened cognitive deficits [33]. Adult rats fed alcohol for 5 weeks had increased levels of APP and BACE1 in several brain regions and increased presenilin-1 and nicastrin in the hippocampus [34]. Long-term alcohol consumption significantly impaired spatial memory in adult rats, which may be a contributing factor to development of AD [87]. These findings show that alcohol increases amyloidogenic processing, a mechanism which could contribute to plaque burden. However, it is not known if increases in plaque numbers were dependent on reduced microglial activities (e.g., phagocytosis) which otherwise could compensate for increased Aβ production.

The current findings have several limitations, a primary one being that these studies were done using enriched cultures of primary rat microglia, which differ from acutely isolated brain microglia in terms of gene expression and function. It is therefore important that analogous studies be carried out to test the effects of alcohol consumption on the microglial transcriptome, and on amyloid phagocytosis, in a transgenic mouse model of amyloid deposition. Second, we only evaluated effects of a single acute exposure to ethanol, which likely will differ from effects following chronic exposure. Since alcohol consumption in humans can involve periods of consumption followed by periods of withdrawal, it is important to determine how withdrawal influences microglial gene expression and phagocytosis.

## Conclusion

These results define the changes that occur to microglial cells following exposure to physiologically relevant amounts of ethanol. While changes in inflammatory mRNAs were expected, observations that alcohol induces changes in mRNAs involved in phagocytosis including members of the complement system is a novel finding suggesting that alcohol consumption can lead to dysregulation of clearance processes in the CNS. In vitro results showing reduced uptake of Aβ, together with the finding that alcohol increase Pycard (ASC) expression which can play a role in amyloid plaque formation, have important implications for AD patients as well as those at risk to develop disease.

## Additional file

Additional file 1: **Table S1.** Differentially expressed mRNAs due to alcohol treatment “Alc-DEs”. Shading indicates mRNA levels were confirmed by qPCR. **Table S2** Differentially expressed mRNAs due to TII treatment “TII-DEs”. **Table S3** Differentially expressed mRNAs due to alcohol and TII treatment “AlcTII-DEs”. **Table S4** Treatment-group overlap of differentially expressed mRNAs. Grey, involved in immune effector responses; Blue, mitochondrial ribosomal subunit; Orange, nuclear ribosomal subunit. **Table S5** KEGG pathways identified in treatment groups. **Table S6** FPKM (fragments per kilobase of transcript per million fragments mapped). (ZIP 3000 kb)

## Abbreviations

AD: Alzheimer’s disease
Alc*-DEs: DEs induced by alcohol or alcohol plus TII, but not by TII alone
Alc-DEs: DEs induced by alcohol treatment
AlcInf-DEs: DEs only induced by alcohol plus TII treatment
AlcOnly-DEs: DEs only induced by alcohol alone
AlcTII-DEs: DEs induced by alcohol plus TII treatment
AMY: Amygdala
APP: Amyloid precursor protein
DE: Differentially expressed
DID: Drinking in the dark
DMEM: Dulbecco’s modified Eagle’s media
ERCC: External RNA Controls Consortium
FACS: Flow activated cell sorting
FAM: 6-Carboxyfluorescein
GO: Gene ontology
IFNγ: Interferon gamma
IL1β: Interleukin 1-beta
NAC: Nucleus accumbens
PFC: Prefrontal cortex
qPCR: Quantitative reverse-transcription PCR
RNAseq: RNA sequencing
ROS: Reactive oxygen species
TII: Mixture of IL1β, IFNγ, and TNFα
TII-DEs: DEs induced by TII treatment
TNFα: Tumor necrosis factor alpha
